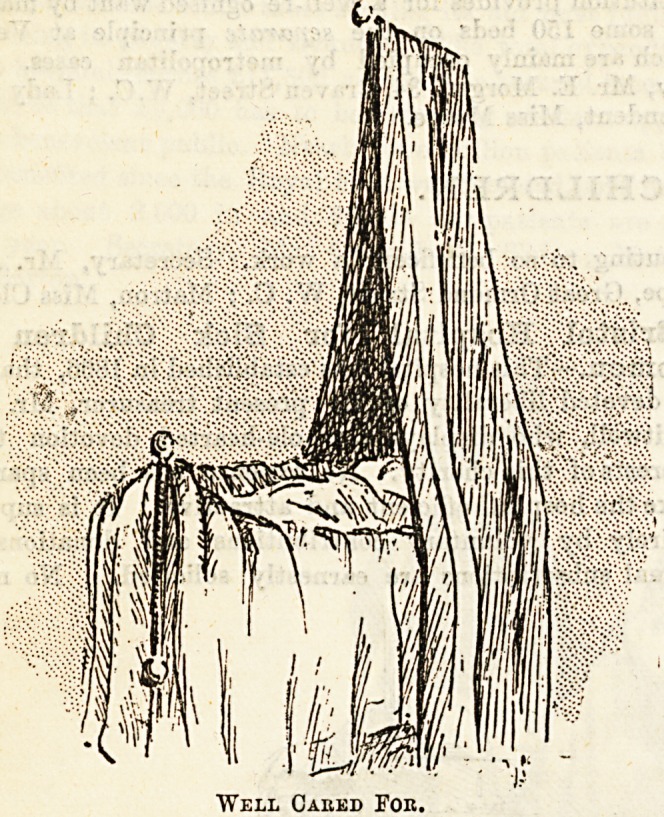# Lying-In Hospitals

**Published:** 1891-01-03

**Authors:** 


### LYING-IN HOSPITALS.

**City of London Lying-In Hospital,** City Road, E.C.—This hospital, situated in a poor and crowded district,

Well Cared For.

is one that should appeal to all wives and mothers, its aim being to offer skilled treatment and care to poor women in their hour of greatest need. The benefits of this institution are doubled by the fact that not only does it receive as many cases within its walls as space will permit, but it serves as a training-school for midwives who attend those in their own homes who cannot gain admittance. Including the out and in-patients relieved by means of this institution, above 1,900 women received its bounty in the past year. Even after leaving the hospital, its kind influence is felt by the assistance offered by the Samaritan Fund, a branch of the charity which offers relief to such poor patients who may be destitute of necessaries and want the means of procuring needful comforts for themselves and their infants. We hope that the public will recognise with generosity this most invaluable institution. Secretary, Mr. R. A. Owthwaite; Matron, Miss S. E. Allen.

**British Lying-In,** Endell Street, W.C.—Secretary, Mr. Fitz Roy Gardner; Matron, Mrs. E. Freeman.

**East End Mothers' Lying-in Home,** Juniper Street, Shadwell, E.—As its name implies, this unpretending but most useful charity has done an infinity of good, over seven hundred patients having been treated since the opening in 1884. Secretary and Lady Superintendent, Mrs. Ashton Warner.

**Royal Maternity Charity,** 31, Finsbury Square.—About four thousand poor women are assisted annually. Secretary, Mr. J. W. Long.

## Figures and Tables

**Figure f1:**